# Psychometric validation of the Chinese version of the PROMIS Social Relationships Short Forms

**DOI:** 10.1002/nop2.1077

**Published:** 2021-09-26

**Authors:** Tingting Cai, Qingmei Huang, Fulei Wu, Haozhi Xia, Changrong Yuan

**Affiliations:** ^1^ School of Nursing Fudan University Shanghai China; ^2^ School of Nursing Nanjing University of Chinese Medicine Nanjing China

**Keywords:** breast cancer, patient‐reported outcomes, psychometric validation, social relationships

## Abstract

**Aim:**

This study aimed to culturally adapt and psychometrically validate the PROMIS Social Relationships Short Forms (PROMIS‐SR) among Chinese patients with breast cancer.

**Design:**

A cross‐sectional study.

**Method:**

The PROMIS‐SR was translated into simplified Chinese by strictly adhering to the Functional Assessment of Chronic Illness Therapy translation method and was subsequently tested among patients with breast cancer (*N* = 965). Eligible patients filled out the demographic information questionnaire, the PROMIS‐SR, and the Functional Assessment of Cancer Therapy‐Breast.

**Results:**

Confirmatory factor analysis (CFA) provided support for the original structure of the PROMIS‐SR. All instruments reported strong known‐group, cross‐cultural and convergent validity, as hypothesized. Correlation coefficients ranged from 0.67 to 0.85, and Cronbach's α of all items were high (0.90–0.94).

## 1 | INTRODUCTION

Breast cancer is the most frequently occurring cancer among women worldwide. Patients with breast cancer require constant care and support from others. Social health is a critical domain of breast cancer care related to clinical health outcomes (Paladino et al., 2019). However, cancer‐related issues such as perceived low body image and frequent hospital visits among survivors of breast cancer might lead to social isolation and deteriorated relationships (Oh et al., 2019). The cancer treatments for breast cancer are protracted and include surgery, chemotherapy, radiation and other treatments in different combinations. Therefore, frequent hospital visits and completion of cancer treatments several times per month significantly restructure their social relationships, profoundly impact patients’ involvement in social activities, fulfil their social roles and result in fewer opportunities to obtain support from others. Evidence indicates that the reassurance from family members, friends, colleagues and health personnel is the most important form of support for patients with breast cancer (Drageset et al., 2012). It has been reported that patients with breast cancer are more likely to underuse their support networks and received less support after cancer treatment, especially in the first year of their treatment trajectory (Chang et al., 2019). Therefore, the start of cancer treatment marks the beginning of an increasing tendency of new unmet social relationship needs. Socially isolated patients who lack access to care, especially from social networks, are more likely to suffer from increased risk of mortality and negative outcomes after breast cancer diagnosis due to poor emotional and mental well‐being (Fong et al., 2017). For women with breast cancer, inadequate social relationships are associated with increased depressive symptoms, poorer quality of life, and more symptom burden (Beasley et al., 2010; Kroenke et al., 2016). Feelings of social isolation and loss of intimacy require strong social relationships and support during their treatment and survivorship to shift (Oh et al., 2019).

Support from patient's surroundings contributes to positive health outcomes (Chang et al., 2019). Patients with breast cancer who have insufficient social relationships tend to report poor psychological well‐being at higher rates (Zamanian et al., 2020). High‐level social relationships enable patients to gain a positive perspective on their medical condition and strengthen their ability to fight breast cancer (Flannery et al., 2019; Ozdemir and Tas Arslan, 2018). Therefore, an accurate evaluation of social relationship needs can enable nurses to modify their course of treatment and ensure that they adequately address their patient's requirements (Huang et al., 2020; Sakai et al., 2019). Despite the important role those social relationships from statistically significant persons play for patients with breast cancer, few of the available validated comprehensive measures assess the social relationships from the patient's perspective (Cai et al., 2020).

Patient perspectives of their health status provide valuable information for medical decision‐making. Patient‐reported outcomes (PROs) assess patients’ experience and priorities and may thereby promote individual care provision (Bevans et al., 2014). Incorporating PROs into clinical settings is especially helpful in situations where there are no clear clinical variables to understand patients’ health status.

Patient‐Reported Outcomes Measurement Information System (PROMIS) supports the increased usage of PROs in guiding clinical decisions (Cella et al., 2007, 2010). PROMIS comprises a set of globally generalizable measures, which efficiently collects self‐reported data from the general population and those with enduring illnesses about symptoms and functions (Brandon et al., 2017; Yount et al., 2019). By acknowledging the important role those social relationships play, and the lack of assessment tools, social relationships are regarded as a primary component of the PROMIS social health framework (Hanmer et al., 2017). The social relationships component is measured by social support and isolation concepts (Hanmer et al., 2017). The original English version of the PROMIS Social Relationships Short Forms (PROMIS‐SR) was developed using mixed methods and administered to several large, diverse samples with varied clinical problems (Hanmer et al., 2017). The PROMIS‐SR contains domains of emotional, informational and instrumental support, assessing expressions of being loved, esteemed, valued, and cared for, tangible aid and service, and the support of advice, suggestions, and information, respectively (Hahn et al., 2014). The measure allows clinicians to assess a person's self‐reported social relationship needs using a minimal number of items without losing the precision of a longer measure (Carlozzi et al., 2018).

## 2 | BACKGROUND

The English version of the PROMIS‐SR has shown adequate reliability and validity results and has been translated into other language versions such as the Spanish version (Hahn et al., 2014; Carlozzi et al., 2019). However, the effectiveness of the instruments in the Chinese population lacks substantial evidence. Research suggests that Asians are more likely to be concerned about seeking social support and afraid their disclosure might have a negative effect on the social relationships with others (Hsu et al., 2020). China has a large sample of women with breast cancer, with a mean age of 45–55 years at the diagnosis of breast cancer, which is considerably younger than Western patients (Fan et al., 2014). It has been reported that young patients were more likely to be vulnerable to cancer treatment (Miyashita et al., 2015). Considering Chinese patients with breast cancer are vulnerable to unmet social relationships needs, this study aimed to adapt PROMIS‐SR culturally and linguistically, and to perform a psychometric validation of the instruments among Chinese patients with breast cancer.

## 3 | THE STUDY

### 3.1 | Design

This study employed a cross‐sectional design, which involved translation and cognitive interviews followed by a psychometric evaluation.

### 3.2 | Method

#### 3.2.1 | Phase 1: Translation and Cognitive Interview Procedure

**FIGURE 1:**
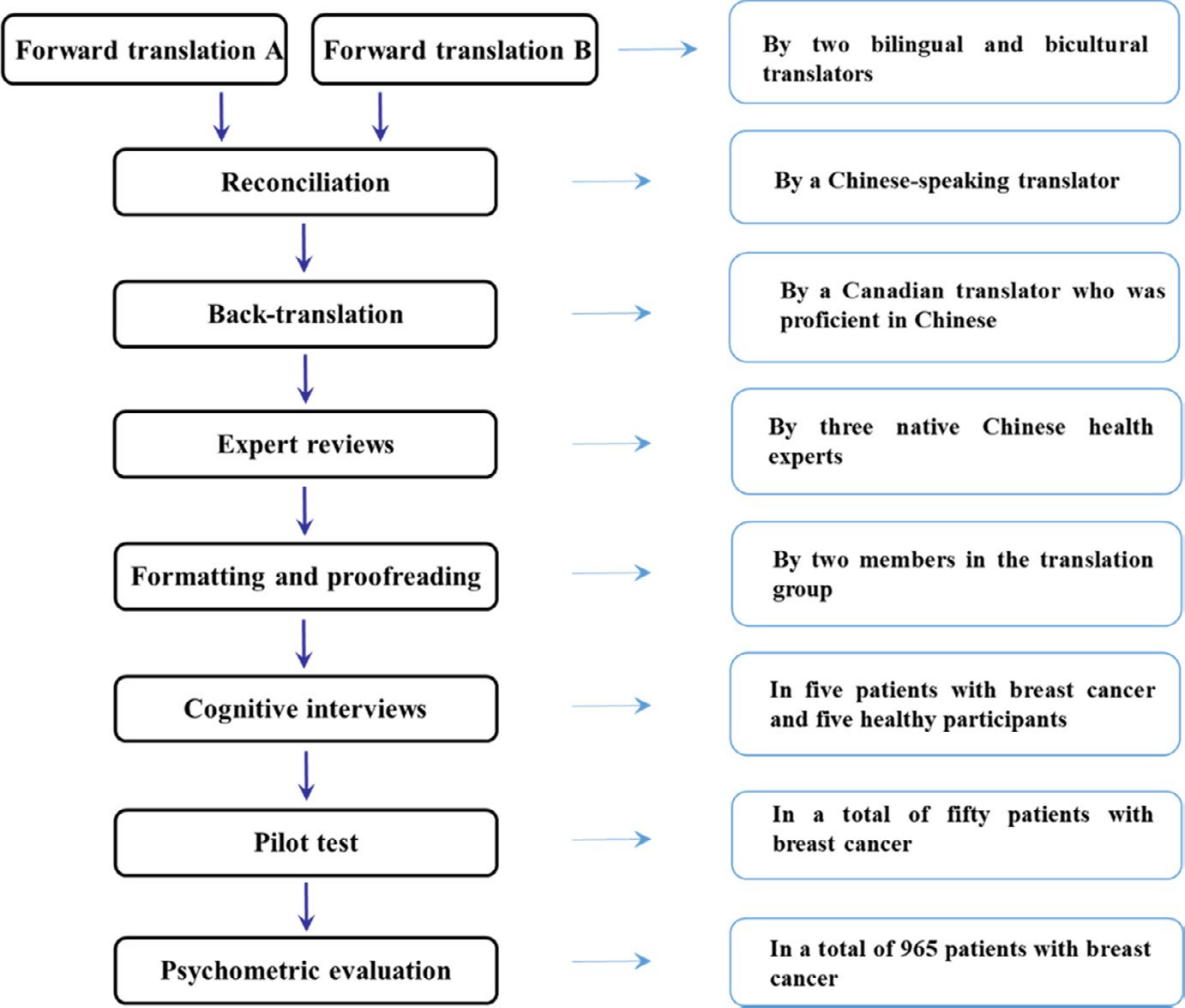
Flow of the phases of the study

The English version of the PROMIS‐SR was translated into simplified Chinese by strictly adhering to the translation method of the Functional Assessment of Chronic Illness Therapy (Irwin et al., 2009; Wild et al., 2009). Figure 1 presents the flow of the translation procedure, where bilingual teams from Canada and China were involved in forward translations, reconciliation, back‐translation and expert reviews.

The translators involved in this procedure followed these inclusion criteria:

1) Educational qualification of master's degree or higher;

2) Linguistic and healthcare experts;

3) Working as Chinese–Canadian nursing researchers or researchers who are native Chinese speakers.

Once the translators reached a consensus about the final version of the translated PROMIS‐SR, cognitive interviews were conducted among native Chinese‐speaking adolescents to evaluate their interpretation and response to each item in the PROMIS‐SR. The PROMIS‐SR are brief and accurate instruments for assessing different types of social relationships among patients with enduring disease and the general population. Therefore, both patients with breast cancer and the general population were included in the cognitive interview. These interviews were conducted with five patients with breast cancer and five healthy participants for each item. Furthermore, participants were asked to reframe the items that were confusing or difficult to understand. The PROMIS‐SR was later modified, pilot‐tested and revised with samples of patients with breast cancer (*N* = 50).

#### 3.2.2 | Phase 2: Psychometric Evaluation

##### Settings and participants

Data were collected from patients with breast cancer in tertiary hospitals of the Shanghai province between January 2018 and September 2020. Trained research nurses reviewed hospital medical records to identify eligible patients using purposive sampling. The inclusion criteria for recruiting patients were: 1) female, 2) received a diagnosis of breast cancer, 3) received medical treatment for breast cancer, 4) aged 18 years or older, and 5) able to speak, read and write Mandarin Chinese. Patients with mental disorders or critical conditions were excluded from this study. Socio‐demographic data and the questionnaires were self‐reported by the patients. On the contrary, clinical data were obtained and reviewed from their medical records by trained nursing researchers. After given written consent to participate, the patients completed the demographic information questionnaire, the PROMIS‐SR, and the Functional Assessment of Cancer Therapy‐Breast.

#### 3.2.3 | Measures

##### Demographic information questionnaire

Demographic information was collected from participants, using questionnaires inquiring participants’ age, religious affiliation, marital status, children, educational background, current employment, monthly family income and stage of cancer treatment.

##### PROMIS Social Relationships Short Forms

The original English version of the PROMIS‐SR, including the PROMIS Emotional, Informational, and Instrumental Support Short Forms (4 items each), was validated in this study. All items were answered using a 5‐point Likert‐type scale (1 = *never*, 2 = *rarely*, 3 = *sometimes*, 4 = *usually*, 5 = *always*) (Gottlieb & Bergen, 2010; Hahn et al., 2014). Each short form was separately scored, ranging from 4 to 20, where higher scores indicated better perceived social relationships (Hahn et al., 2010). These obtained scores were then transformed to T‐scores to enable comparison with the national norm (US general population: Mean [M] (T‐score) = 50, Standard Deviation [*SD*] (T‐score) = 10). The PROMIS‐SR has been translated and psychometrically validated in different languages with satisfactory results (Cai et al., 2020; Hahn et al., 2014; Jones et al., 2019). This study included the translation and validation of its Chinese version.

##### Functional Assessment of Cancer Therapy‐Breast (FACT‐B)

The convergent validity of the PROMIS‐SR was tested using the Chinese version of FACT‐B. It includes a general subscale on cancer with domains of physical, social, emotional, and functional well‐being (FACT‐G) and a subscale designed specifically for patients with breast cancer (BCS) (Cella et al., 1993). All items were rated on a 5‐point Likert‐type scale (0 = *not at all*, 1 = *a little bit*, 2 = *somewhat*, 3 = *quite a bit*, and 4 = *very much*). The total score was computed as the sum of all subscale scores, where higher scores indicated higher degrees of quality of life (Algamdi & Hanneman, 2018). The Chinese version of the FACT‐B had been adequately validated among Chinese patients with breast cancer (Ng et al., 2011; Wan et al., 2007).

### 3.3 | Ethics

This study was approved by the ethics committee of the institutional review boards Fudan University and all study sites (number: 1810192–22). The patients were invited to participate in the survey with the help of their nurses during hospitalization. The data were collected by trained nurse researchers in the Department of Breast Surgery from each study site. The patients were informed about the purpose, necessity, process, confidentiality, and voluntary participation requirements of the study prior to the interviews by trained nurse researchers. Signed informed consent was obtained from all patients before completing the questionnaires.

### 3.4 | Analysis

The SPSS Statistics 23 for Windows, Mplus 7.4 and AMOS version 23.0 were used to analyse the obtained data. Demographic characteristics, clinical characteristics, and scale scores were presented using descriptive statistics, wherein categorical and continuous variables were described using frequency, mean values, and standard deviations.

Additionally, CFA was performed to evaluate the factorial validity of the PROMIS‐SR. The ratio of cases to variables was 965:12, which was higher than the recommended rule of thumb value (5–10:1). The sample sizes were sufficient to perform a stable model estimation by CFA (Hu & Bentler, 1999). Model fit was examined using the acceptable criteria as follows: Chi‐Square/Degrees of Freedom (χ^2^/*df* <3), comparative fit index (CFI >0.90), goodness‐of‐fit index (GFI >0.90), Tucker‐Lewis index (TLI >0.90), and Root Means Square Error of Approximation (RMSEA <0.08) (Hu & Bentler, 1999).

Items with Differential item functioning (DIF) indicated that respondents with the same trait from groups with different age, education background, gender have differed probabilities of giving certain response to items (DeWalt et al., 2007). Gender was not examined by the DIF values because the sample did not include male patients. Therefore, DIF across scores of patients of different ages and educational backgrounds were used to assess the cross‐cultural validity of the PROMIS‐SR. Spearman correlation coefficients were used to assess the convergent validity (r < 0.3: negligible, r = 0.3–0.7: acceptable) (Castro‐Rodrigues et al., 2018). Studies reported that employed patients with breast cancer have better social relationships than those who are unemployed (Paalman et al., 2016; Tamminga et al., 2012). We hypothesized that employed patients would report better scores than those who were unemployed. Therefore, known‐group validity was assessed by comparing T‐scores of patients with different employment statuses using a one‐way ANOVA test.

The reliability of the PROMIS‐SR was determined using Cronbach's α coefficient (> 0.70: acceptable), Guttman's Split‐half coefficient (> 0.70: acceptable), and item‐to‐total correlations (r > 0.30: acceptable), where higher values indicate higher item reliability (Castro‐Rodrigues et al., 2018). Statistical significance was set at *P* <0.05.

## 4 | RESULTS

### 4.1 | Characteristics of the participants

Table 1 presents the descriptive statistics for the demographic and clinical characteristics of the participants. Of the 1,072 eligible patients, 51 patients disagreed to participate due to cancer treatment or family reasons. In addition, 56 participants’ data were discarded due to missing responses. Therefore, 965 patients with breast cancer (response rate =90.02%) were analysed. All respondents were aged between 23 and 77 years (*M*±*SD* =49.03 ± 10.34). The data showed that 92.5% of the respondents were married, 97.0% had children, 87.8% were without religious affiliation, 32.5% had received secondary school education, 63.5% were unemployed, 52.3% had a monthly family income of less than ¥6,000 (approximately $900). As for clinical information, 66.6% had crossed the stage of chemotherapy treatment and most of them were in stage Ⅱ and Ⅲ of cancer.

**TABLE 1:** Socio‐demographic and medical information

Variables	*N* (%)
Age (Mean ± *SD*)	49.03 ± 10.34
Marital status	
Single	21 (2.2)
Married	893 (92.5)
Divorced	23 (2.4)
Widowed	28 (2.9)
Children	
Yes	936 (97.0)
No	29 (3.0)
With religious affiliation	
Yes	118 (12.2)
No	847 (87.8)
Education background	
Primary school or below	260 (27.0)
Secondary school	314 (32.5)
High school	191 (19.8)
University or above	200 (20.7)
Current employment	
Employed	154 (16.0)
Retired	198 (20.5)
Unemployed	613 (63.5)
Monthly family income	
≤ ¥ 6000/$900	460 (47.7)
> ¥ 6000/$900	505 (52.3)
Treatment stage	
Chemotherapy	643 (66.6)
Breast cancer surgery	322 (33.4)
Cancer stage	
Ⅰ	133 (13.8)
Ⅱ	283 (29.3)
Ⅲ	255 (26.4)
Ⅳ	114 (11.8)
Not yet determined	180 (18.7)

### 4.2 | Validity analyses

#### 4.2.1 | Factorial validity

**FIGURE 2:**
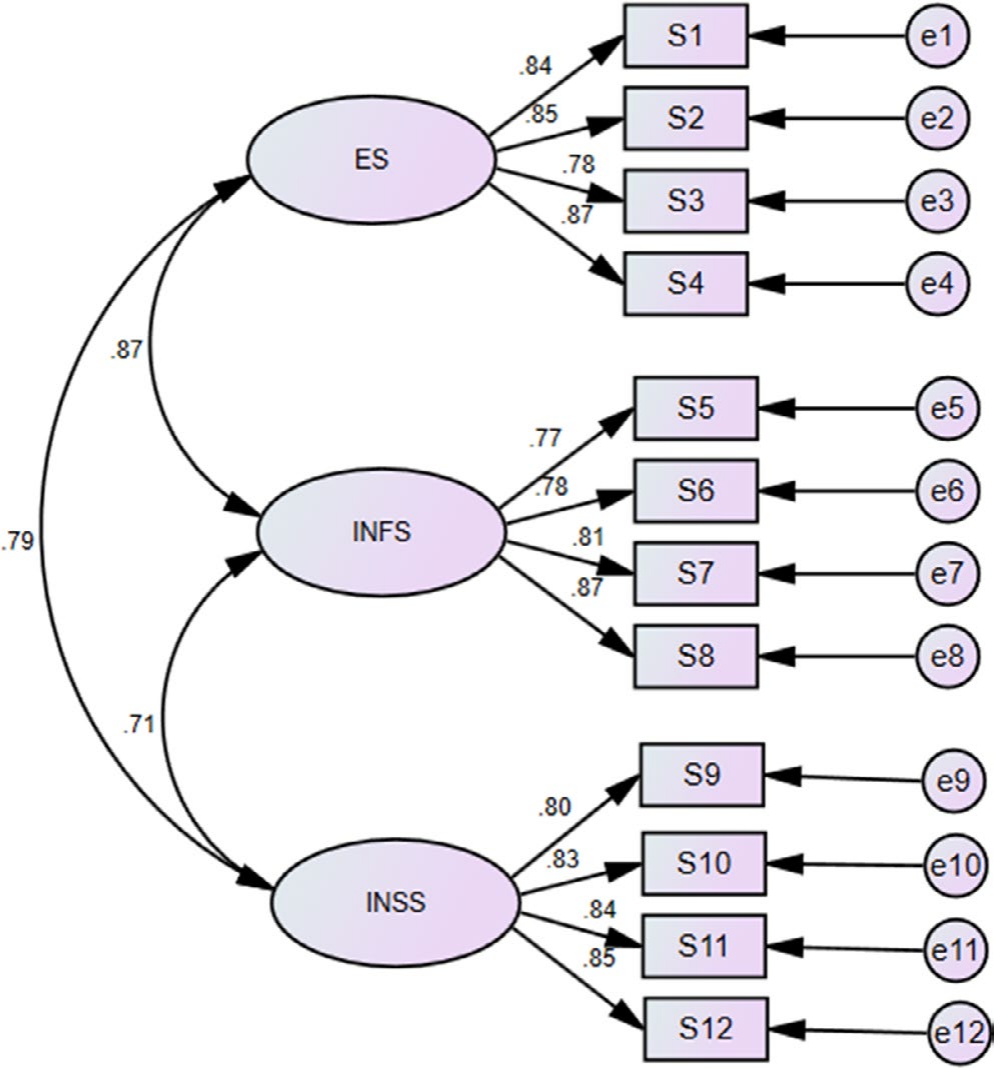
Confirmatory factor analysis model of the PROMIS Social Relationships Short Forms

The factorial validity of the instruments was tested by performing a three‐factor CFA model (Figure 2). The model fit of the data were acceptable: χ^2^/*df* =2.133, *p* <.05, GFI =0.920, CFI =0.926, TLI =0.931, RMSEA =0.038). All factor loadings were high (ranging from 0.77 to 0.87), which indicated statistically significant correlations among the items. These findings provided evidence for a good model fit of the PROMIS‐SR and provided support for the original structure.

#### 4.2.2 | Cross‐cultural validity

In the current study, age (18–39, 40–59, and 60+ years) and educational backgrounds (below primary, secondary, and high school levels, above and below university‐level education) were the two socio‐demographic variables tested for DIF. Table 2 indicates that no statistically significant DIF values were found for differences in items according to age and educational background. This finding suggested that the PROMIS‐SR provided unbiased results across patients of different ages and educational backgrounds.

**TABLE 2:** Differential item functioning among demographic groups of the PROMIS Social Relationships Short Forms

Items	DIF test *P*‐value	
	Age group	Education group
Emotional Support 01	0.319	0.116
Emotional Support 02	0.420	0.872
Emotional Support 03	0.333	0.182
Emotional Support 04	0.524	0.332
Informational Support 01	0.179	0.092
Informational Support 02	0.389	0.369
Informational Support 03	0.428	0.255
Informational Support 04	0.274	0.304
Instrumental Support 01	0.261	0.086
Instrumental Support 02	0.197	0.641
Instrumental Support 03	0.160	0.394
Instrumental Support 04	0.258	0.524

#### 4.2.3 | Convergent validity

Spearman correlation coefficients reported moderate correlations between the PROMIS‐SR and FACT‐B. The highest correlation emerged between the PROMIS Informational Support and the FACT‐B (r = 0.49, *p* <.05), followed by the PROMIS Emotional Support and FACT‐B (r = 0.44, *p* <.05), and lastly, the PROMIS Instrumental Support (r = 0.40, *p* <.05). Therefore, these results provided evidence for the convergent validity of the PROMIS‐SR.

### 4.3 | Known‐group validity

In line with the hypothesis, the mean PROMIS‐SR T‐scores were significantly different between the employed and the unemployed group (*p* <.05) (Table 3). Employed group patients reported higher scores on social relationships than those in the unemployed group. This result suggested that the instruments have an acceptable known‑group validity (Table 3).

**TABLE 3:** Known‑groups validity of the PROMIS Social Relationships Short Forms in patients based on employment status

Known‐group	PROMIS Emotional Support		PROMIS Informational Support		PROMIS Instrumental Support	
	Mean (*SD*)	*P*	Mean (*SD*)	*P*	Mean (*SD*)	*P*
Employed patients	52.49 (8.86)		52.66 (8.89)		52.54 (9.56)	
Unemployed patients	48.74 (10.62)	< 0.05	47.73 (10.79)	< 0.05	48.39 (10.43)	< 0.05

### 4.4 | Reliability analyses

The PROMIS‐SR demonstrated acceptable Cronbach's α and Guttman Split‐half coefficients above the standard of 0.70 (Table 4). All item scores were significantly correlated with their underlying domain scores. The item‐to‐total correlations of PROMIS‐SR (r = 0.67–0.85) indicated a strong item correlation.

**TABLE 4:** Reliability of the PROMIS Social Relationships Short Forms

Short form	Cronbach's alpha	Guttman Split‐half coefficient	Item‐to‐total correlations (Range)
PROMIS Emotional Support Short Form	0.92	0.90	0.67–0.82
PROMIS Informational Support Short Form	0.93	0.90	0.70–0.85
PROMIS Instrumental Support Short Form	0.94	0.92	0.76–0.85

## 5 | DISCUSSION

Measures of social relationships play an important role in emphasizing how patients’ social environments influence their health status. To the best of our knowledge, this is the first study to conduct a translation and psychometric validation of the PROMIS‐SR in the Chinese population of enduring diseases. Additionally, the study evaluates the PROMIS‐SR in a vulnerable population—patients with breast cancer, aiming to aid nurses to satisfy the patients’ unmet social relationship needs. Findings from this work provided evidence for the reliability and validity of the PROMIS‐SR in this population.

The CFA results provided evidence for the factorial validity of the PROMIS‐SR. There was no evidence for DIF concerning age and educational background; this indicates that all items were unbiased measures and reported satisfactory cross‐cultural validity. These findings were consistent with a previous study, which suggested that the English version of the PROMIS‐SR did not report any local dependency on gender, age, education, and ethnicity among adults in the US (Hahn et al., 2014). Furthermore, our results revealed that these instruments reported satisfactory known‐group validity among patients with breast cancer by distinguishing social relationships among patients with different employment statuses. All domains in the PROMIS‐SR demonstrated statistically significant group differences, which in line with the literature, reported that unemployed patients with breast cancer had more social relationship impairments (Paalman et al., 2016; Tamminga et al., 2012). Moreover, moderate Spearman correlation coefficients between T‐scores of the PROMIS‐SR and FACT‐B were consistent with our research hypothesis. Therefore, the convergent validity was supported, as the measure displayed expected correlations with other measure focussing on social health.

Carlozzi et al. (2018) examined the validity of the PROMIS‐SR in caregivers of patients with traumatic brain injury, showing that the measure demonstrated adequate convergent validity with their sample, which was not against our results. Cronbach's α coefficients—the most frequently used indicator of reliability—met a priori criteria of internal consistency (α > 0.70) for the PROMIS‐SR. This finding was supported by similar studies reporting adequate reliability of the measure (Carlozzi et al., 2018; Shensa et al., 2020). Carlozzi et al., (2018) reported strong correlations among the domains of PROMIS‐SR, with correlations ranging from 0.6 to 0.8, which was similar to our findings (r = 0.67–0.85).

The distribution of the PROMIS‐SR scores in our sample of patients with breast cancer is similar to that in the general population in which the measure was developed. However, the mean values for the scores are higher in our sample. Jones et al. (2019) adapted the original English version of PROMIS‐SR. According to the study, 2,988 respondents aged 65 to 69 years completed the PROMIS‐SR in a multi‐site prospective cohort study conducted in the US. The mean T‐scores for the PROMIS Emotional, Informational, and Instrumental Support were 54.83, 56.56 and 57.08, respectively, which was higher than the mean T‐scores in our current study. An explanation for this might be the differences in the characteristics of the samples. It is also possible that the inclusion of patients who were willing to participate might belong to groups with better social relationships than those who refused to participate. Another potential explanation may be the age difference. Most of the patients who participated in the study were middle‐aged women with an average age of 49 years. Breast cancer and its treatment might cause more challenges for social relationships of young women with breast cancer than their middle‐aged counterparts and would be at greater risk for social relationship impairments (Miyashita et al., 2015). However, young women only accounted for a small proportion of the sample in this study. We did not explore the test–retest reliability of the measure because social relationships are a diversified construct.

Therefore, future studies need to confirm these results with larger samples of patients with breast cancer to examine: 1) the need for more language translation and revision, and 2) the social relationships of patients with breast cancer with different age and treatment characteristics.

### 5.1 | Limitations

This study had certain limitations. First, the current study sample may not be entirely representative of all Chinese patients with breast cancer because they were recruited from only three tertiary hospitals, and most of them were middle‐aged. Second, the sample only included female patients due to the lack of male patients suffering from breast cancer. Thus, future studies should include patients with different age groups and male patients to ensure the generalizability of these results. Additionally, the current study followed a cross‐sectional design and did not evaluate test–retest reliability. However, our findings provide a clinical foundation for using the Chinese version of the PROMIS‐SR among different populations and settings.

## 6 | CONCLUSION

An accurate and brief social relationship needs evaluation may enable nurses to screen patients who require tailored social support. The current study findings provided evidence of adequate reliability and validity of the Chinese version of the PROMIS‐SR to perform routine assessments of social relationship needs among Chinese patients with breast cancer. Future studies are needed to draw more firm conclusions about its applicability.

## CONFLICT OF INTEREST

The authors declare that there is no conflict of interest.

## AUTHOR CONTRIBUTIONS

TC and CR: Study design, data analysis and wrote manuscript. TC, QH and CR: Analysed and interpreted the data. FW and HX: Acquired the data, revised the manuscript for important intellectual content. All authors read and approved the final manuscript.

## DATA AVAILABLILITY STATMENT

Data are available upon reasonable request.
